# SpaMWGDA: Identifying spatial domains of spatial transcriptomes using multi-view weighted fusion graph convolutional network and data augmentation

**DOI:** 10.1371/journal.pcbi.1013667

**Published:** 2025-11-12

**Authors:** Lin Yuan, Boyuan Meng, Qingxiang Wang, Chunyu Hu, Cuihong Wang, De-Shuang Huang

**Affiliations:** 1 Key Laboratory of Computing Power Network and Information Security, Ministry of Education, Shandong Computer Science Center, Qilu University of Technology (Shandong Academy of Sciences), Jinan, China; 2 Shandong Engineering Research Center of Big Data Applied Technology, Faculty of Computer Science and Technology, Qilu University of Technology (Shandong Academy of Sciences), Jinan, China; 3 Shandong Provincial Key Laboratory of Industrial Network and Information System Security, Shandong Fundamental Research Center for Computer Science, China; 4 The Second Qilu Hospital of Shandong University, Jinan, China; 5 Ningbo Institute of Digital Twin, Ningbo Key Laboratory of Multi-Omics & Multimodal Biomedical Data Mining and Computing, Eastern Institute of Technology, Ningbo, China; 6 Collaborative Innovation Center (2011) for Medical and Health Big Data of Guizhou Province, School of Biology and Engineering (School of Modern Industry for Health and Medicine), Guizhou Medical University, Guiyang, China; Shanghai Institute of Nutrition and Health, Chinese Academy of Sciences, CHINA

## Abstract

The rapid development of spatial transcriptomics (ST) has made it possible to effectively integrate gene expression and spatial information of cells and accurately identify spatial domains. A large number of deep learning (DL)-based methods have been proposed to perform spatial domain identification and achieved impressive results. However, these methods have some limitations. First, these methods rely on a fixed similarity metric and cannot fully utilize neighborhood information. Second, they cannot efficiently and adaptively integrate key information when fusing and reconstructing gene expression using purely additive methods. Finally, these methods ignore key nonlinear features and introduce noise during clustering. To address these limitations, we propose a novel DL model SpaMWGDA based on multi-view weighted fused graph convolutional network (GCN) and data augmentation. By modeling spatial information using different similarity metrics, the model is able to successfully capture comprehensive neighborhood information of the spot features. By combining data augmentation and contrastive learning, SpaMWGDA is able to learn key gene expressions. SpaMWGDA uses a multi-view GCN encoder to model the similarities between spatial information and gene features, and uses a view-level attention mechanism for weighted fusion to adaptively learn the dependencies between them and learn the key features of each view. Experimental results not only demonstrate that SpaMWGDA outperforms competing methods in spatial domain identification and trajectory inference but also show the ability of SpaMWGDA to analyse tissue structure and function. The source code for SpaMWGDA is available at https://github.com/nathanyl/SpaMWGDA .

## Author summary

DL-based methods have some limitations. First, these methods rely on a fixed similarity metric and cannot fully utilize neighborhood information. Second, they cannot efficiently and adaptively integrate key information when fusing and reconstructing gene expression using purely additive methods. Finally, these methods ignore key nonlinear features and introduce noise during clustering. We propose a novel DL model SpaMWGDA based on multi-view weighted fused graph convolutional network (GCN) and data augmentation. SpaMWGDA uses a multi-view GCN encoder to model the similarities between spatial information and gene features, and uses a view-level attention mechanism for weighted fusion to adaptively learn the dependencies between them and learn the key features of each view. Experimental results not only demonstrate that SpaMWGDA outperforms competing methods in spatial domain identification and trajectory inference but also show the ability of SpaMWGDA to analyse tissue structure and function.

## Introduction

Spatial transcriptomics (ST) technologies [1–5] can pinpoint gene expression while maintaining the structural integrity of tissues, helping us to understand molecular communication and tissue structure. Spatial domains are areas with similar spatial gene expression distribution.

Precise identification of the spatial domain is a crucial step in ST data analysis. It helps to elucidate the complex relationships between gene expression, its spatial characteristics, and tissue functions, and also aids in understanding the distribution and interactions of cells within the tissue structure [6–8]. However, accurately identifying spatial domains remains challenging due to the frequent presence of loss and noise in the generated data. Many computational methods have been developed for spatial domain identification. The shallow learning algorithms (e.g., K-means, Louvain [9] and Leiden [10]) are often used in Scanpy [11] or Seurat [12] packages to form integrated analysis workflows. The spatial domains identified by these methods are usually discontinuous because they ignore the adjacent relationships between spatial domains [13]. Giotto [14] identified spatial domains by comparing the intrinsic gene expression patterns of neighboring cells using hidden Markov random field (HMRF). The method is computationally complex and difficult to deal with large-scale datasets. In addition, HMRF may have limitations in capturing nonlinear relationships between cells. BayesSpace [15], based on a fully Bayesian statistical approach, encouraged neighboring cells to belong to the same group via predefined spatial priors. However, BayesSpace fails to effectively utilize spatial coordinates, and the choice of spatial priors may affect the clustering results.

Recently, deep learning (DL)-based spatial clustering methods have emerged as powerful tools for spatial domain identification [16–24]. For example, stlearn [25] efficiently performed spatial domain identification by integrating gene expression, spatial location, and tissue morphology. spaGCN [26] employed an undirected weighted graph to represent spatial data dependencies and incorporated spatial location, histological images, and gene expression into the graph’s construction to identify spatial domains. DeepST [27] used a denoising autoencoder and a graph neural network (GNN) autoencoder to infer latent embeddings in enhanced ST data. However, noise and inaccurate spot relationships in histological images may lead to erroneous spatial domain identification results. To better utilize high-resolution ST data, researchers considered the spatial dependencies of gene expression by modeling the similarity between neighboring spots. For example, SEDR [28] used a deep autoencoder and a variational graph autoencoder to integrate transcriptomics data with relevant spatial information. STAGATE [29] employed a graph attention autoencoder to integrate spatial information and gene expression, learning latent representations to distinguish spatial domains. GraphST [30] combined GNNs with self-supervised contrastive learning to identify spatial domains by learning spot representations. Although these methods can identify spots as distinct regions, they cannot adaptively capture the interrelationships between gene expression and spatial information. Recently, multi-view graph convolutional network (GCN)-based methods have been proposed to learn the relationship between spatial location and gene expression more effectively [31]. For example, Spatial-MGCN [32] utilized a multi-view GCN encoder to identify spatial domains. STMGCN [33] introduced an unsupervised learning framework that learns view-specific spot representations and integrates them with an attention mechanism to generate the final representation of the spots. But multi-view convolutional networks encounter several challenges, including noise and inconsistent feature distributions across views, which can hinder effective fusion and compromise stability. Additionally, certain views may contribute minimally, and inappropriate fusion strategies may introduce redundancy, further affecting model performance.

Although these methods have achieved remarkable results, there are still some limitations. First, these methods rely on a fixed similarity metric and cannot fully utilize neighborhood information. Second, they cannot efficiently and adaptively integrate key information when fusing and reconstructing gene expression using purely additive methods. Finally, these methods ignore key nonlinear features and introduce noise during clustering [34,35].

In this article, we are interested in integrating multi-view weighted fusion graph convolutional network (GCN) and data augmentation to guide deep learning architecture to accurately identify spatial domains. Multi-view Graph Convolutional Networks enhance the analytical accuracy and generalization capabilities of spatial transcriptomics data by integrating data from multiple perspectives to augment the model’s expressive power. The weighted fusion attention mechanism dynamically adjusts feature weights to identify critical regions or genes, thereby improving prediction precision. Data augmentation enhances model robustness by generating new samples, particularly in scenarios with sparse or imbalanced data, further boosting generalization performance.

**Fig 1:**
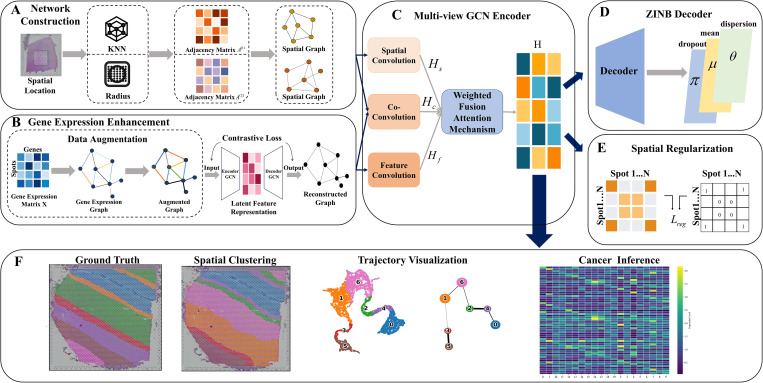
Schematic overview of SpaMWGDA. **(A)** Spatial neighborhood network construction module. **(B)** Gene expression enhancement module. **(C)** Multi-view weighted fusion GCN encoder. **(D)** ZINB decoder. **(E)** Spatial regularization constraint. **(F)** Downstream analysis.

Here, we name the proposed spatial domain identification method as SpaMWGDA. The workflow of SpaMWGDA is presented in Fig 1. First, SpaMWGDA employs KNN and Radius to construct a neighborhood graph [36,37], which can help the model successfully capture the comprehensive neighborhood information of point features. Second, SpaMWGDA learns key feature representations from original and augmented gene expression data by constructing graph-embedded contrastive encoder [38,39]. Third, SpaMWGDA integrates a multi-view GCN encoder and a ZINB (Zero-Inflated Negative Binomial) decoder [40,41] to reconstruct the gene expression matrix. In multi-view GCN encoder, the weighted fusion attention mechanism dynamically adjusts the weight of each view, enabling the model to better adapt to the contribution of different views, thus improving model performance. The view-level attention mechanism can adaptively and effectively integrate information from multiple views, thereby improving the performance and robustness of the model. Finally, SpaMWGDA uses a spatial regularization constraint to train the model to cluster neighboring points in space and effectively separates spatially non-neighboring points. We compared the performance of SpaMWGDA with seven state-of-the-art methods on five datasets (three from 10 x Visium, one from Stereo-seq platform and one from Xenium). Experimental results not only indicate that SpaMWGDA outperforms seven state-of-the-art methods in spatial domain identification and trajectory inference but also demonstrate the ability of SpaMWGDA to analyse tissue structure and function.

## Results

### Ablation experiments

To explore the contribution of key modules to the performance of SpaMWGDA, we constructed five variants of SpaMWGDA and performed ablation experiments on DLPFC datasets. The five variant models are: (i) (w/o)Radius; (ii)(w/o)KNN;(iii) (w/o)CL; (iv) (w/o)WFA and (v) (w/o)KNN and Radius. (w/o)Radius represents that SpaMWGDA only uses KNN (without Radius) to identify neighboring points. (w/o)KNN represents that SpaMWGDA only uses Radius (without KNN) to identify neighboring points. (w/o)CL represents that SpaMWGDA only use original gene expression to obtain feature representation without using contrastive learning. (w/o)WFA represents that SpaMWGDA uses equal weights instead of dynamic weights in the attention layer. (w/o)KNN and Radius represents that SpaMWGDA uses a distance-based similarity matrix to model spatial information.

**Fig 2:**
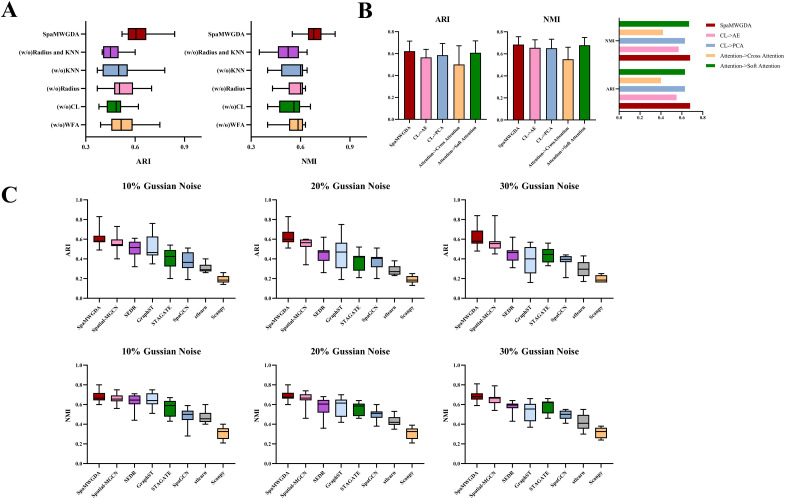
(A) Ablation experiment results. **(B)** The performance of contrastive learning and weighted fusion attention on SpaMWGDA. **(C)** The results of SpaMWGDA and seven competing methods on the noisy DLPFC dataset.

As shown in Fig 2A, compared to SpaMWGDA, the ARI and NMI of (w/o)Radius, (w/o)KNN, (w/o)CL, (w/o)WFA and (w/o)KNN and Radius decreased by 16.1% and 16.2%, 17.7% and 17.6%, 21% and 20.6%, 16.1% and 19.1%, and 37.1% and 17.6%, respectively. The ARI and NMI of each module were listed in Table 1. The experimental results highlight the importance of constructing spatial graphs using different similarity metrics, contrastive learning using augmented gene features, and using weighted fusion attention mechanisms. These modules can help the model better identify the interaction between spatial information and genetic features, improve the accuracy of spatial domain identification, and ultimately improve model performance.

**Table 1:** The ARI and NMI of four variant models and SpaMWGDA.

	(w/o)WFA	(w/o)CL	(w/o)Radius	(w/o)KNN	(w/o)KNN and Radius	SpaMWGDA
**ARI**	0.60	0.61	0.54	0.57	0.5	0.73
	0.54	0.45	0.37	0.38	0.4	0.66
	0.52	0.50	0.50	0.50	0.45	0.67
	0.51	0.49	0.47	0.48	0.42	0.53
	0.75	0.62	0.70	0.78	0.6	0.55
	0.60	0.52	0.60	0.65	0.5	0.52
	0.47	0.47	0.62	0.47	0.45	0.62
	0.52	0.50	0.51	0.51	0.5	0.84
	0.45	0.38	0.50	0.50	0.5	0.60
	0.39	0.39	0.37	0.37	0.4	0.57
	0.48	0.48	0.55	0.50	0.45	0.61
	0.40	0.42	0.48	0.38	0.4	0.57
**AVG.**	0.52	0.49	0.52	0.51	0.39	0.62
**NMI**	0.63	0.60	0.60	0.60	0.6	0.78
	0.59	0.55	0.43	0.49	0.49	0.72
	0.62	0.64	0.62	0.62	0.62	0.69
	0.40	0.45	0.63	0.64	0.64	0.66
	0.63	0.58	0.55	0.60	0.6	0.55
	0.54	0.40	0.52	0.48	0.48	0.60
	0.53	0.59	0.57	0.60	0.6	0.73
	0.60	0.61	0.63	0.63	0.63	0.81
	0.58	0.53	0.60	0.60	0.6	0.68
	0.40	0.43	0.49	0.40	0.4	0.64
	0.59	0.57	0.60	0.55	0.55	0.68
	0.53	0.54	0.60	0.45	0.45	0.67
**AVG.**	0.55	0.54	0.57	0.56	0.56	0.68

### The performance of contrastive learning and weighted fusion attention on model

In order to further evaluate the impact of contrastive learning and weighted fusion attention on the model, we constructed four variants of SpaMWGDA: (i) CL- > PCA; (ii) CL- > AE [42], (iii) Attention- > Cross Attention; and (iv) Attention- > Soft Attention [43]. Principal component analysis (PCA) and autoencoder (AE) are widely used feature learning methods. CL- > PCA indicates using PCA instead of contrastive learning. and CL- > AE replaces the contrastive learning with autoencoder. The weighted fusion attention mechanism is replaced by the cross-attention mechanism and the soft attention mechanism respectively to evaluate the impact of the weighted fusion attention mechanism on SpaMWGDA.

We compared the performance of these four variants with SpaMWGDA on the DLPFC and human breast cancer datasets. As shown in the Fig 2B, SpaMWGDA outperforms these variants in terms of ARI and NMI. Contrastive learning enables SpaMWGDA to learn critical and discriminative nonlinear features. The adaptive property of the weighted fusion attention mechanism is particularly beneficial in integrating information from different views, helping to extract features that are critical for spatial clustering, thereby improving the performance of the model.

PCA is a linear dimensionality reduction method that cannot capture nonlinear relationships. Autoencoder is effective in learning latent representations, but may focus too much on global features during reconstruction and ignore subtle differences between cell types that are critical for spatial clustering. Cross attention mechanism uses multiple attentions, which increases computational complexity and may affect model performance. Soft attention mechanism assigns weights to input features to focus on important information. However, it may over-rely on global features and ignore local details.

### The impact of noise on model robustness and the scalability of SpaMWGDA

To assess the impact of noise on the performance of SpaMWGDA, we constructed three kinds of random gaussian noise datasets based on the DLPFC dataset: (i) Gaussian Noise (10%); (ii) Gaussian Noise (20%); (iii) Gaussian Noise (30%). Gaussian Noise (10%), Gaussian Noise (20%), and Gaussian Noise (30%) respectively represent adding 10%, 20%, and 30% Gaussian noise to the original gene expression data, respectively. We compared the performance of SpaMWGDA with state-of-the- art methods (Scanpy, stlearn, SpaGCN, SEDR, STAGATE, GraphST, and Spatial-MGCN) on three noisy datasets. As shown in Fig 2C, although the performance of SpaMWGDA decreases due to the increase of noise, it still outperforms the competing methods. The results of SpaMWGDA and seven competing methods on the Gaussian Noise 10% DLPFC dataset were listed in Table 2, and the results of the remaining noise dataset were listed in S1 Table. These results showed that SpaMWGDA is robust to noise and its performance remains excellent even as noise increases.

**Table 2:** The ARI and NMI of SpaMWGDA and seven competing methods on Gaussian Noise 10% DLPFC dataset.

ARI	SpaMWGDA	Spatial-MGCN	STAGATE	SEDR	GraphST	SpaGCN	stLearn	Scanpy
151507	0.74	0.66	0.43	0.49	0.45	0.47	0.40	0.23
151508	0.65	0.54	0.47	0.49	0.52	0.35	0.34	0.26
151509	0.59	0.53	0.36	0.61	0.43	0.39	0.40	0.25
151510	0.50	0.56	0.42	0.46	0.45	0.33	0.34	0.21
151669	0.57	0.44	0.24	0.44	0.35	0.23	0.28	0.14
151670	0.49	0.40	0.20	0.32	0.42	0.19	0.28	0.16
151671	0.58	0.61	0.49	0.56	0.65	0.46	0.29	0.18
151672	0.83	0.73	0.53	0.54	0.76	0.51	0.27	0.14
151673	0.59	0.56	0.54	0.58	0.65	0.47	0.26	0.21
151674	0.57	0.54	0.36	0.59	0.48	0.33	0.29	0.19
151675	0.58	0.55	0.31	0.56	0.56	0.30	0.30	0.16
151676	0.57	0.53	0.49	0.38	0.45	0.38	0.26	0.16
**AVG.**	0.61	0.55	0.40	0.50	0.51	0.37	0.31	0.19
**NMI**	**SpaMWGDA**	**Spatial-MGCN**	**STAGATE**	**SEDR**	**GraphST**	**SpaGCN**	**stLearn**	**Scanpy**
151507	0.77	0.75	0.59	0.63	0.66	0.54	0.52	0.36
151508	0.72	0.67	0.63	0.61	0.64	0.47	0.53	0.36
151509	0.64	0.67	0.59	0.70	0.61	0.53	0.60	0.40
151510	0.65	0.65	0.59	0.62	0.64	0.49	0.50	0.33
151669	0.62	0.57	0.47	0.56	0.51	0.40	0.42	0.21
151670	0.60	0.56	0.43	0.44	0.56	0.28	0.41	0.24
151671	0.71	0.74	0.64	0.67	0.75	0.53	0.46	0.29
151672	0.80	0.70	0.66	0.66	0.74	0.58	0.40	0.24
151673	0.68	0.64	0.67	0.71	0.73	0.59	0.45	0.38
151674	0.65	0.66	0.50	0.71	0.63	0.44	0.43	0.32
151675	0.66	0.65	0.45	0.67	0.67	0.45	0.46	0.33
151676	0.67	0.63	0.60	0.60	0.60	0.51	0.44	0.28
**AVG.**	0.68	0.66	0.57	0.63	0.65	0.48	0.47	0.31

We calculated the running time of SpaMWGDA under different data scales and spot counts. The results in Table 3 show the scalability of SpaMWGDA on large-scale datasets.

**Table 3:** Running time of SpaMWGDA under different data scales and spot counts.

Data Scale	10%	20%	30%	40%	50%
Running Time(minute)	6mins	12mins	18mins	24mins	30mins
**Spot Scale**	**10%**	**20%**	**30%**	**40%**	**50%**
Running Time(minute)	2mins	5mins	8mins	10mins	12mins

### Performance comparison of methods for identifying spatial domain

To evaluate the performance of SpaMWGDA in spatial domain identification, we conducted a comparison with seven state-of-the-art methods: Scanpy, stlearn, SpaGCN, SEDR, STAGATE, GraphST, and Spatial-MGCN, using the 10x Visium DLPFC dataset, which contains 12 slices [44]. These seven methods include two shallow learning methods (Scanpy, stlearn) and five state-of-the-art DL-based methods (SpaGCN, SEDR, STAGATE, GraphST, and Spatial-MGCN).

**Fig 3:**
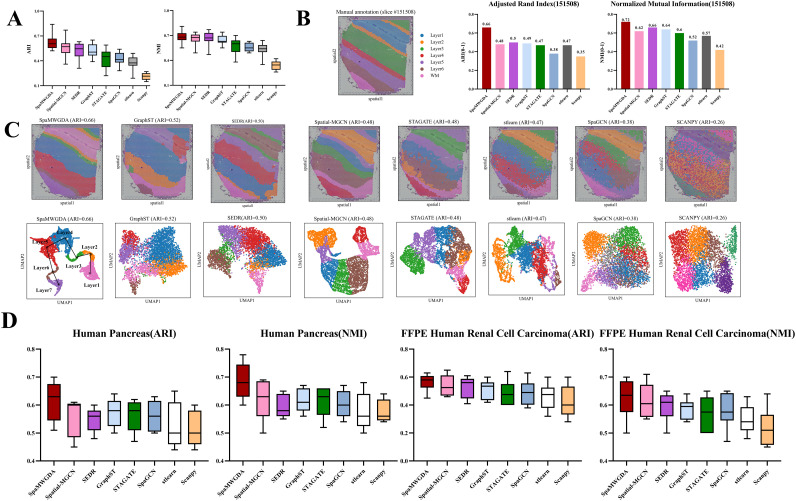
(A) The performance comparison of spatial domain identification of SpaMWGDA and seven state-of-the-art methods (Scanpy, stlearn, SpaGCN, SEDR, STAGATE, GraphST, and Spatial-MGCN) on DLPFC dataset. **(B)** Comparison of ARI and NMI of SpaMWGDA with seven state-of-the-art methods (Scanpy, stlearn, SpaGCN, SEDR, STAGATE, GraphST, and Spatial-MGCN) on DLPFC slice 151508. **(C)** The performance comparison of spatial domain identification of SpaMWGDA and seven state-of-the-art methods (Scanpy, stlearn, SpaGCN, SEDR, STAGATE, GraphST, and Spatial-MGCN) on DLPFC slice 151508. **(D)** The performance comparison of SpaMWGDA and seven state-of-the-art methods on Human Pancreas dataset and FFPE Human Renal Cell Carcinoma dataset.

As shown in Fig 3A, SpaMWGDA achieves the best clustering across these slices, with the highest mean ARI of 0.62 and the highest mean NMI of 0.68, surpassing Spatial- MGCN, SEDR, STAGATE and GraphST by 0.06 and 0.02, 0.1 and 0.01, 0.13 and 0.05, and 0.1 and 0.04, respectively. The mean ARI and mean NMI of other methods (Scanpy, stlearn and SpaGCN) are below 0.45 and 0.6, respectively. Detailed results for all 12 slices in the DLPFC dataset were shown in S1 Fig. It is important to note that while Scanpy exhibits minimal variation in ARI between slices, its median NMI is only 0.35. In contrast, the performance of Spatial-MGCN and STAGATE shows instability, with significant ARI variation across slices. The mean values of three histology-informed spatial clustering methods (stlearn and SpaGCN) are all below 0.45, which indicates that histological images may introduce noise that adversely affects clustering performance.

To further validate the effectiveness of SpaMWGDA, we show the identification results of DLPFC slice 151508. As shown in Fig 3B, compared to competing methods, SpaMWGDA achieves a better fit with the ground truth, with ARI and NMI of 0.66 and 0.72 respectively, while the ARI and NMI of competing methods are all below 0.6 and 0.7 respectively. As shown in Fig 3C, Scanpy shows layer mixing, SEDR shows unclear cluster boundaries, Spatial-MGCN only achieves an ARI of 0.48, GraphST and STAGATE confuse spots from layer 3 and WM (White Matter), and both stlearn and SpaGCN fail to distinguish most DLPFC layers. In UMAP visualization analysis, the clustering divisions of Scanpy and SpaGCN are not clear. Although STAGATE, SEDR, GraphST, and stlearn achieve good clustering results, the hierarchical order is unclear. The embeddings of both SpaMWGDA and Spatial-MGCN clearly show the cortical development trajectories, and the UMAP map of SpaMWGDA not only reveals the different clustering divisions in each domain, but also highlights the clear sequential relationships between layers. These results indicate the excellent clustering performance of SpaMWGDA and highlight the advantage of SpaMWGDA in identifying spatial domains. In addition, we also compared the performance of SpaMWGDA and seven competing methods on the Human Pancreas dataset from 10x Visium HD and FFPE Human Renal Cell Carcinoma dataset from 10x Xenium. The results show that SpaMWGDA is better than other methods.

### SpaMWGDA reveals the laminar structure of the olfactory bulb from Stereo-seq data and infers its biological functions

Stereo-seq technology provides highly detailed spatial and molecular data for spatial transcriptomics studies of the mouse olfactory bulb (MOB) [45], enabling the evaluation of models’ ability to explore biological functions. For comparative studies, we selected Scanpy, GraphST, SEDR, STAGATE, Spatial-MGCN, stlearn, and SpaGCN as benchmark methods to assess the spatial domain identification capability of SpaMWGDA.

**Fig 4:**
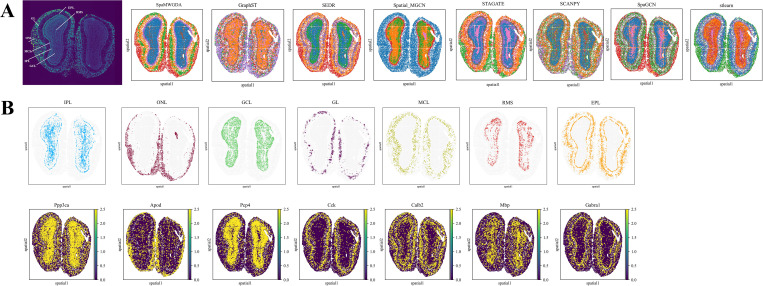
(A) Comparison of the results of identifying the laminar structure of the olfactory bulb using SpaMWGDA and seven state-of-the-art methods (Scanpy, stlearn, SpaGCN, SEDR, STAGATE, GraphST, and Spatial-MGCN). **(B)** Analysis results of differentially expressed genes (DEGs) between the coronal tissue layers of the mouse olfactory bulb identified by SpaMWGDA.

As shown in Fig 4A, Scanpy does not clearly delineate the partitions, and GraphST and Spatial-MGCN confuse different regions. SEDR, stlearn, STAGATE, SpaGCN, and SpaMWGDA all accurately capture the hierarchical structure of the MOB. SEDR divides the region into seven layers. However, it fails to link the identified regions to any specific areas in the histological images and fails to detect the narrow glomerular layer. STAGATE, stlearn and SpaGCN segment the MOB into seven regions, most of which are accurately identified, but there is still considerable overlap between clusters. SpaMWGDA exploits a multi-view weighted fusion GCN encoder to deeply explore the intrinsic relationship between gene expression and spatial information, thereby obtaining more informative and discriminative latent representations.

We then performed a detailed analysis of the differentially expressed genes (DEGs) between the coronal tissue layers of the mouse olfactory bulb identified by SpaMWGDA. As shown in Fig 4B, the results revealed that the identified clusters were strongly correlated with the expression of well-known marker genes such as Apod and Cck [46]. The olfactory nerve layer (ONL) corresponds to the expression domain of the Apod gene, which is involved in encoding components of high-density lipoproteins, suggesting that the ONL may be linked to lipoprotein metabolism. The Cck gene encodes cholecystokinin (CCK), which modulates neuronal excitability and synaptic transmission and plays a key role in the processing and feedback regulation of olfactory signals [47,48]. CCK has an important influence on the glomerular layer (GL), which is responsible for the initial integration of olfactory information. CCK enhances the transmission and perception of olfactory signals by regulating the activity of neurons in this region. These findings not only demonstrate the ability of SpaMWGDA to handle tissue structures at different resolutions, but also indicate that SpaMWGDA can infer potential tissue functions from identified spatial domains, offering valuable insights for further exploration of unknown tissue structures and functions.

### SpaMWGDA delivers detailed insights into tumor heterogeneity in human breast cancer

**Fig 5:**
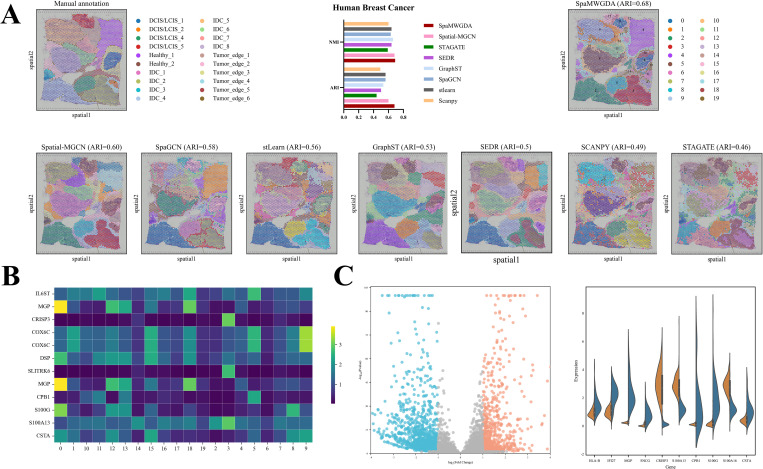
(A) The performance comparison of spatial domain identification of SpaMWGDA and seven state-of-the-art methods (Scanpy, stlearn, SpaGCN, SEDR, STAGATE, GraphST, and Spatial-MGCN) on human breast cancer dataset. **(B)** Results of differentially expressed genes analysis among different clusters identified by SpaMWGDA. **(C)** Results of differentially expressed genes analysis between cluster 3 (IDC) and cluster 13 (DCIS/LCIS).

In this section, we analysed the human breast cancer dataset from 10x Visium platform, which contains 20 domains and 4 main tissue types: DCIS/LCIS, healthy tissues, IDC, and hypomalignant tumor margins. As shown in Fig 5A, SpaMWGDA achieves the highest ARI and NMI among all methods. Compared to competing methods, SpaMWGDA can consistently identify domains that align with manual annotations and accurately detect domains such as Healthy_1 and IDC_5, with smoother boundaries for each domain. In contrast, the boundaries of regions identified by Scanpy are highly irregular and contain a large amount of noise. Although Spatial-MGCN, SEDR, SpaGCN, stlearn, GraphST, and STAGATE identify more domains than Scanpy, these domains still have rough boundaries and outliers.

We further validated the performance of SpaMWGDA in detecting cancer tissue heterogeneity using the human breast cancer dataset. We compared the expression of the top differentially expressed genes (DEGs) in clusters 4 (healthy), 13 (DCIS/LCIS), 3 (IDC), and 15 (tumor margins), and found significant heterogeneity among these clusters (Fig 5B). We also performed differential expression analysis (|logFoldChange| ≥ 2 and P-value < 0.05) between cluster 3 (IDC) and cluster 13 (DCIS/LCIS) to explore gene expression differences between IDC and DCIS/LCIS, and identified about 100 significant DEGs between these two clusters (Fig 5C). As shown in Fig 5C, among the significant differentially expressed genes (DEGs) identified, CRISP3 [49] plays a pivotal role in breast cancer by regulating tumor cell migration, invasion, and immune modulation. It contributes to the tumor microenvironment through inflammatory pathways and immune evasion mechanisms. Additionally, MGP (Matrix Gla Protein), a calcium-binding protein, is integral to extracellular matrix remodeling and mineralization, and its elevated expression is associated with tumor aggressiveness and adverse prognosis. According to the studies [27,50], we found that the upregulation of CRISP3, S100A13, and S100A16 in domain 3 suggests that this region possesses tumor invasiveness, metastatic potential, and an active inflammatory environment, thereby promoting tumor progression. In contrast, domain 13, with high expression of MGP, CPB1, and S100G, points to a region associated with calcification, ECM remodeling, and tumor progression, which may contribute to treatment resistance and poor clinical outcomes.

These findings highlight the critical roles of CRISP3 and MGP in breast cancer pathogenesis, positioning them as valuable prognostic biomarkers and potential therapeutic targets. The distinct molecular profiles observed in domains 3 and 13 underscore the regional heterogeneity of the tumor microenvironment and the need for region-specific therapeutic strategies. In summary, SpaMWGDA offers a more refined approach to dissect cancer tissue heterogeneity and enhances our understanding of ST data.

## Discussion

In this paper, we proposed a novel deep learning model, SpaMWGDA, combined a multi-view weighted fused graph convolutional network and data augmentation for spatial domain identification of spatial transcriptome (ST) data. By leveraging multi-view weighted fusion and data enhancement, SpaMWGDA effectively learns the relationships between spatial information and gene expression. Experimental results demonstrated that SpaMWGDA outperforms competing methods in terms of clustering accuracy and identification of biologically relevant domains, and exhibits superior performance in spatial domain identification. Additionally, SpaMWGDA successfully evaluated ST data from various platforms with different spatial resolutions. Experiment results highlighted the importance of integrating different similarity metrics for exploring spatial information, as well as reconstructing gene expression through data enhancement and weighted fusion attention mechanism.

However, SpaMWGDA still has limitations. For instance, in experiments involving model noise injection, we can employ various noise addition methods such as signal-to-noise ratio (SNR) to validate the model's robustness from different perspectives. When considering the use of different similarity measures to construct spatial neighborhood graphs, we can experiment with alternative metrics, such as the cosine similarity measure based on vectors. Utilizing spatial multi-omics data [51] will enable the model to more accurately resolve the spatial domain, which is vital for inferring the biological functions of complex tissues in organisms. Therefore, spatial domain identification methods that effectively use spatial multi-omics data should be proposed in future work.

## Materials and methods

### Data preparation

To validate the performance of the model, we used five datasets from multiple platforms. As shown in Table 4, the first dataset is from 10 x Visium, with each slice containing five to seven manually annotated regions of the human dorsolateral prefrontal cortex (DLPFC) [44]. The second dataset is the ST dataset of human breast cancer from 10 x Visium, which contains 20 domains and four major morphological types: DCIS/LCIS (Ductal Carcinoma In Situ/Lobular Carcinoma In Situ), IDC (invasive ductal carcinoma), healthy tissues, and hypomalignant tumor margins [52]. The third dataset is a mouse olfactory bulb dataset obtained from Stereo-seq [53], which annotates the RMS (rostral migratory stream), GCL (granular cell layer), IPL (internal plexiform layer), MCL (mitral cell layer), EPL (external plexiform layer), and ONL (olfactory nerve layer). The fourth dataset is a Human Pancreas dataset from 10x Visium HD [54]. It offers spatial gene expression data from human pancreas tissue, enabling analysis of gene activity across different pancreatic regions. The fifth dataset is a FFPE Human Renal Cell Carcinoma dataset from 10x Xenium [55]. It provides high-resolution spatial transcriptomic data from formalin-fixed, paraffin-embedded (FFPE) human renal cell carcinoma tissue. It allows for the exploration of gene expression patterns within the tumor microenvironment, aiding in the study of tumor heterogeneity and cellular interactions in cancer.

**Table 4:** Overview of five ST datasets used in this study.

Datasets	Spots	Genes	Slices	Domains	Platforms
DLPFC	3460-4789	33538	12	5/7	10x Visium
Human Breast Cancer	3798	36601	1	20	10x Visium
Mouse Olfactory Bulb	19109	14376	1	7	Stereo-seq
Human Pancreas	5000-10000	2000-5000	5	5	10x Visium HD
FFPE Human Renal Cell Carcinoma	More than 10000	10000	6	7	10x Xenium

SpaMWGDA takes gene expression and spatial location as input. To reduce technical noise, spots outside the main tissue areas were first removed. Subsequently, SCANPY [11] was used to filter genes with low-expression and low-variance, eliminate genes that were not expressed in less than 100 cells, and select top 3,000 highly variable genes (HVGs). Finally, the expression data of HVGs were normalized to the total expression level of each cell to 10,000. The formula can be defined as follows:


$$x_{ij}^{\prime }=\left(\frac{x_{ij}}{\sum {\mathrm{\backslash nolimits}}_{j}x_{ij}}\right)\times 10000$$ (1)

where $x_{ij}(1\leq i\leq N,1\leq j\leq M)$ represents the j-th gene expression value at the i-th point.

### Experiment settings

For SpaMWGDA, we employed the learning rate of 0.001, the weight decay of 5e-4, and utilized the ADAM optimization algorithm. Additionally, to optimize the nearest neighbor search, we adopted the K-D-tree algorithm and tested various k values ranging from 1 to 20. Performance scores were calculated for each k value, enabling dynamic adjustment of k to enhance the quality of the neighborhood matrix. The radius r is set based on the data resolution. All experiments are repeated 10 times, and spatial domain recognition performance is evaluated using ARI and NMI. The average of 10 runs is taken to obtain a reasonable performance assessment.

### The SpaMWGDA framework

SpaMWGDA extracts both gene expression and spatial location from ST data, leveraging a deep neural network architecture to identify spatial domains. As depicted in the Fig 1, SpaMWGDA consists of five key modules: (i) spatial neighborhood network construction module; (ii) gene expression enhancement module; (iii) multi-view weighted fusion GCN encoder; (iv) ZINB decoder; and (v) spatial regularization constraint.

In the spatial neighborhood network construction module, KNN and Radius similarity measures are used to create a normalized adjacency matrix, ensuring the model adapts to ST data with varying resolutions and fully utilizes neighborhood information. The gene expression enhancement module enhances gene expression data, yielding improved feature representations. A graph-based contrastive encoder is employed to learn effective representations of gene expression from original and enhanced features. This enhancement allows adaptive integration of key information when reconstructing gene expression. The multi-view weighted fusion GCN encoder extracts embeddings from gene expression data, spatial location data, and their combinations. These embeddings are adaptively fused via a view-level attention mechanism, which helps model minimize noise during clustering. Afterwards, the ZINB decoder reconstructs the feature matrix to capture the global information of the spatial expression spectrum. Finally, a spatial regularization constraint is incorporated into the learning process of the model to ensure that spatial neighborhood information is preserved and the inherent spatial structure of the data is maintained.

### Spatial neighborhood networks construction

K-nearest neighbor (KNN) can effectively capture the local structure of data, while Radius helps to uncover the global structure of the data [56]. We construct two undirected neighborhood graphs $G_{s}^{\left(n\right)}=(V,E^{\left(n\right)}),n=1,2$ using KNN and Radius respectively. Here V represent the set of points, and $E^{\left(n\right)}$ represent the set of edges in n-th graph. The adjacency matrix is defined as ${A_{s}}^{\left(n\right)}\in R^{N*N}$, N is the number of points. $A_{ij}^{\left(1\right)}=A_{ji}^{\left(1\right)}=1$ if points i and j are each other’s K-nearest neighbors, and otherwise 0. $A_{ij}^{\left(2\right)}=A_{ji}^{\left(2\right)}=1$ if points i and j are within a radius, and otherwise 0. We employ K-D tree algorithm to determine the best k value and set the radius r according to the data resolution. The normalized adjacency matrix is calculated as follows:


$$A^{\left(n\right)}={\tilde{D}}^{{\left(n\right)}^{-\frac{\text{1}}{\text{2}}}}{{\tilde{A}}_{s}}^{\left(n\right)}{\tilde{D}}^{{\left(n\right)}^{-\frac{\text{1}}{\text{2}}}}$$ (2)

where ${\tilde{A}}_{s}^{\left(n\right)}={A_{s}}^{\left(n\right)}+I_{N}$, ${\tilde{D}}^{\left(n\right)}=\sum_{j}^{N}{\tilde{A}}_{ij}^{\left(n\right)}$, *n* = 1,2, which ${{\tilde{A}}_{s}}^{\left(n\right)}$ is an adjacency matrix with additional self-connections, $I_{N}$ is a unit matrix.

### Gene expression enhancement

1) Feature graph construction: SpaMWGDA calculates gene expression similarity using cosine distance. $G_{f}=(A_{f},X)$ is graph of the gene expression matrix X, $A_{f}\in R^{N*N}$ is the feature adjacency matrix, $A_{f}^{ij}=1$ if point j is the nearest neighbor of point i, and otherwise 0.
2) Data augment: We perform contrastive learning [57] on gene expression to learn latent representations from both original and augmented data. SpaMWGDA takes $\left(A_{f},X\right)$ as input and extracts spot features without changing graph structure. For contrastive learning, we generate corrupted neighborhood graph $\left(A_{f},X^{\prime }\right)$ on the $\left(A_{f},X\right)$, which scrubs gene expression data while maintaining the original neighborhood graph topology.
3) Graph-embedded contrastive encoder: We develop a graph-embedded contrastive encoder to learn key feature representations. The model consists of three modules: graph convolutional encoder, graph deconvolutional decoder, and deep contrastive self-encoder. The process is summarized as follows:
  - Graph convolutional encoder: We utilize a GCN as the encoder to extract relevant feature representations from both original and augmented data.

$$H_{0}^{\left(l\right)}=\phi_{0}^{\left(l\right)}(W_{0}^{\left(l\right)}H_{0}^{\left(l-1\right)}*p_{c}(L)+b_{0}^{\left(l\right)})$$ (3)

$$H_{1}^{\left(l\right)}=\phi_{1}^{\left(l\right)}(W_{1}^{\left(l\right)}H_{1}^{\left(l-1\right)}*p_{c}(L)+b_{1}^{\left(l\right)})$$ (4)

The normalized Laplacian matrix is denoted as $L=I-D^{-\frac{\text{1}}{\text{2}}}AD^{-\frac{\text{1}}{\text{2}}}$, and D represents the degree matrix. The activation functions $\phi_{0}^{\left(l\right)}$ and $\phi_{1}^{\left(l\right)}$ for the l-th GCN encoder layer are applied to X and $X^{\prime }$, respectively. The weight matrices of the encoder for X and $X^{\prime }$ are denoted as $W_{0}^{\left(l\right)}$and $W_{1}^{\left(l\right)}$, respectively. The bias terms for the VAE corresponding to X and $X^{\prime }$ are represented as $b_{0}^{\left(l\right)}$ and $b_{1}^{\left(l\right)}$. In this model, we use the convolutional operator of GCN as the convolution operation. For convenience, we refer to the gene expression matrices X and $X^{\prime }$ as $H_{0}^{\left(0\right)}$ and $H_{1}^{\left(0\right)}$, respectively.

- Graph deconvolutional decoder: Although graph convolution is adept at capturing local feature, the resulting smoothing can adversely affect the quality of data reconstruction and compromise the learnable global features. We adopt a graph deconvolution network (GDN) as a decoder to alleviate the negative effects of graph convolution and learn feature representation more effectively.

$$H_{0}^{\left(k\right)}=\varphi_{0}^{\left(k\right)}(W_{0}^{\left(k\right)}H_{0}^{\left(k-1\right)}*p_{d}(L)+b_{0}^{\left(k\right)})$$ (5)

$$H_{1}^{\left(l\right)}=\phi_{1}^{\left(k\right)}(W_{1}^{\left(k\right)}H_{1}^{\left(k-1\right)}*p_{d}(L)+b_{1}^{\left(k\right)})$$ (6)

where $\varphi_{0}^{\left(k\right)}$ and $\phi_{1}^{\left(k\right)}$ are the activation functions of the k-th GDN decoder layer for X and $X^{\prime }$, respectively. $W_{0}^{\left(k\right)}$ and $W_{1}^{\left(k\right)}$ denote the weight matrix of decoder for X and $X^{\prime }$, respectively. $b_{0}^{\left(k\right)}$ and $b_{1}^{\left(k\right)}$ correspond to the bias term of VAE for X and $X^{\prime }$, respectively. And the deconvolutional operator $p_{d}$ is the inverse function of $p_{c}$.

- Deep contrastive self-encoder: To effectively balance local and global information, we propose a deep contrastive learning strategy as a constraint for feature learning. This approach enhances SpaMWGDA’s ability to represent complex information. The loss of contrastive learning can be expressed as:

$$L_{com}=\frac{\text{1}}{N}\sum_{i=1}^{N}\left[sim(f_{orig}(A_{f},X),f_{aug}(A_{f},X^{\prime }))-sim(f_{orig}(A_{f},X),f_{neg}(A_{f},X^{\prime }))+\alpha \right]$$ (7)

where $f_{orig}(A_{f},X)$ and $f_{aug}(A_{f},X^{\prime })$ represent the feature representation on original graph $\left(A_{f},X\right)$ and corrupted graph $\left(A_{f},X^{\prime }\right)$ respectively, and α controls the distance difference between positive and negative samples.

### Multi-view weighted fusion GCN encoder

GCN is a powerful neural network that processes graph data by aggregating information from neighboring nodes, capturing dependencies, and generating embeddings [58]. We utilize a multi-view weighted fusion GCN encoder to extract important information [33]. This encoder consists of four main modules: spatial convolution, feature convolution, co-convolution, and weighted fusion attention mechanism.

1) Spatial convolution: A convolution operation is performed on to aggregate spatial information of the neighbors and multi-layer spatial convolutional network applies hierarchical propagation rules:

$$\{\begin{array}{c} {}*20cZ_{s}^{\left(l+1\right)}=ReLU\mathrm{\backslash nolimits}({\tilde{D}}_{s}^{-\frac{\text{1}}{\text{2}}}{\tilde{A}}_{s}{\tilde{D}}_{s}^{-\frac{\text{1}}{\text{2}}}Z_{s}^{\left(l\right)}W_{s}^{\left(l\right)})\\ Z_{s\_a}^{\left(l+1\right)}=ReLU\mathrm{\backslash nolimits}({\tilde{D}}_{s\_a}^{-\frac{\text{1}}{\text{2}}}{\tilde{A}}_{s\_a}{\tilde{D}}_{s\_a}^{-\frac{\text{1}}{\text{2}}}Z_{s\_a}^{\left(l\right)}W_{s\_a}^{\left(l\right)}) \end{array}$$ (8)

where $W_{s}^{\left(l\right)}$, $W_{s\_a}^{\left(l\right)}$ are weight matrices of l-th layer in spatial convolution, and initially $Z_{s}^{\left(0\right)}=Z_{s\_a}^{\left(0\right)}=X$. $D_{s}$ and $D_{s\_a}$ are diagonal matrices of $A_{s}$ and $A_{s\_a}$ respectively. ${\tilde{A}}_{s}=A_{s}+I,{\tilde{A}}_{s\_a}=A_{s\_a}+I$, and the joint embedding is $Z_{s}^{\left(l+1\right)}=\frac{Z_{s}^{\left(l+1\right)}+Z_{s\_a}^{\left(l+1\right)}}{\text{2}}$.

2) Feature convolution: To learn more comprehensive gene expression information from latent representations obtained by contrast learning, feature convolution is performed on $A_{f}$ and $X^{\prime }$:

$$Z_{f}^{\left(l+1\right)}=ReLU\mathrm{\backslash nolimits}({\tilde{D}}_{f}^{-\frac{\text{1}}{\text{2}}}{\tilde{A}}_{f}{\tilde{D}}_{f}^{-\frac{\text{1}}{\text{2}}}Z_{f}^{\left(l\right)}W_{f}^{\left(l\right)})$$ (9)

where $W_{f}^{\left(l\right)}$ is weight matrix of l-th layer, initially $Z_{f}^{\left(0\right)}=X^{\prime }$.

3) Co-convolution: We adopt a parameter sharing strategy to extract co-embedding of gene expression and spatial distribution:

$$\{\begin{array}{c} {}*20cZ_{sc}^{\left(l+1\right)}=ReLU\mathrm{\backslash nolimits}({\tilde{D}}_{s}^{-\frac{\text{1}}{\text{2}}}{\tilde{A}}_{s}{\tilde{D}}_{s}^{-\frac{\text{1}}{\text{2}}}Z_{sc}^{\left(l\right)}W_{c}^{\left(l\right)})\\ Z_{sc\_a}^{\left(l+1\right)}=ReLU\mathrm{\backslash nolimits}({\tilde{D}}_{s\_a}^{-\frac{\text{1}}{\text{2}}}{\tilde{A}}_{s\_a}{\tilde{D}}_{s\_a}^{-\frac{\text{1}}{\text{2}}}Z_{s\_ac}^{\left(l\right)}W_{c}^{\left(l\right)})\\ Z_{fc}^{\left(l+1\right)}=ReLU\mathrm{\backslash nolimits}({\tilde{D}}_{f}^{-\frac{\text{1}}{\text{2}}}{\tilde{A}}_{f}{\tilde{D}}_{f}^{-\frac{\text{1}}{\text{2}}}Z_{fc}^{\left(l\right)}W_{c}^{\left(l\right)}) \end{array}$$ (10)

where $W_{c}^{\left(l\right)}$ is weight matrix of l-th layer, and initially $Z_{sc}^{\left(0\right)}=Z_{s\_ac}^{\left(0\right)}=Z_{fc}^{\left(0\right)}=X^{\prime }$. $Z_{c}^{\left(l+1\right)}=\frac{Z_{sc}^{\left(l+1\right)}+Z_{s\_ac}^{\left(l+1\right)}+Z_{fc}^{\left(l+1\right)}}{\text{3}}$ is joint embedding. The normalization constraint is defined as follows:


$$L_{con}={\left\|{\tilde{Z}}_{sc}{{\tilde{Z}}^{T}}_{sc}-{\tilde{Z}}_{s\_ac}{{\tilde{Z}}^{T}}_{s\_ac}\right\|}_{2}^{2}+{\left\|{\tilde{Z}}_{sc}{{\tilde{Z}}^{T}}_{sc}-{\tilde{Z}}_{fc}{{\tilde{Z}}^{T}}_{fc}\right\|}_{2}^{2}$$ (11)

### Attention mechanism

In order to adaptively learn the importance of each latent embedding, we introduce a weighted attention mechanism. The final attention weight is obtained by calculating weighted sum of attention weights of each view.


$$att\_weight=Softmax\mathrm{\backslash nolimits}(Linear(W_{2}*\tanh(W_{1}*x_{i}+b_{1})+b_{2}))$$ (12)

where $x_{i}$ is the feature representation of each view, $x_{i}\in \left\{Z_{s},Z_{c},Z_{f}\right\}$, $W_{1}$, $W_{2}$, $b_{1}$, $b_{2}$ are weights and biases.

The weighted fusion module performs weighted fusion on the features of different views according to the weights to obtain an integrated feature representation. The weighted fusion is computed as follows:


$$att_{x_{i}}=att\_weight_{x_{i}}*x_{i},x_{i}\in \left\{Z_{s},Z_{c},Z_{f}\right\}$$ (13)

The weighted merged features are accumulated into the final integrated feature representation$Z=\sum_{i=1}^{n}att_{x_{i}}$.

### ZINB decoder

The ZINB decoder is widely used to reconstruct gene expression matrix to capture the global information [59]. The latent low-dimensional representation Z is used as the input. Given gene expression data X of the ST data, $\pi_{ij},\mu_{ij},\theta_{ij},b_{ij}$ are parameter matrices of zero-inflation parameter, mean, decoder output discretization, and bias vector, respectively. The decoder outputs the estimated values of three parameters.


$$\{\begin{array}{c} {}*20cp_{nb}(x_{ij}|b_{i})=\frac{\Gamma (x_{ij}+\theta_{ij})}{\Gamma (x_{ij}+1)\Gamma (\theta_{ij})}{\left(\frac{\theta_{ij}}{\theta_{ij}+\mu_{ij}}\right)}^{\theta_{ij}}{\left(\frac{\mu_{ij}}{\theta_{ij}+\mu_{ij}}\right)}^{x_{ij}}\\ p_{zinb}(x_{ij}|b_{i})=ZINB(x_{ij}|\pi_{ij},\mu_{ij},\theta_{ij},b_{i})=0+(1-\pi_{ij})p_{nb}(x_{ij}|b_{i})\\ L_{zinb}=-\sum_{j=1}^{N}\sum_{i=1}^{N}\left[\log\pi_{ij}p_{zinb}\left(x_{ij}|b_{i}\right)\right] \end{array}$$ (14)

### Spatial regularization constraint

Spatially neighboring points should be close to each other, while spatially non-neighboring points should be far apart in the latent space [60–62]. Similarity information and spatial neighborhood information are used to compute the loss of spatial regularization constraint:


$$L_{reg}=-\frac{\text{1}}{\text{2}}(\frac{\text{1}}{\left|\varepsilon \right|}\sum_{(i,j)\in R_{i}}\log(\sigma (sim_{ij}))+\frac{\text{1}}{\left|N\right|}\sum_{(i,j)\notin R_{i}}\log(1-\sigma (sim_{ij})))$$ (15)

where $sim_{ij}$ represents the cosine similarity between embedding vector $x_{i}$ of i-th point and embedding vector $x_{j}$ of j-th point in learning-based potential representation H. ε represents the set of all neighboring nodes of point i, N represents the set of all non-neighboring nodes of point i, and $R_{i}$ represents the set of spatial neighbors of point i.

SpaMWGDA learns more informative and discriminative latent representations by maximizing the similarity of neighboring point pairs and minimizing the similarity of non-neighboring point pairs.

### Evaluation strategies

We compared SpaMWGDA with Scanpy [11], stlearn [25], SpaGCN [26], SEDR [28], STAGATE [29], GraphST [30], and Spatial-MGCN [32] to test the performance of SpaMWGDA. These seven methods include two shallow learning algorithms (Scanpy, stlearn) and five state-of-the-art DL-based methods (SpaGCN, SEDR, STAGATE, GraphST, and Spatial-MGCN). Scanpy (2018) is a Python library that provides data processing, dimensionality reduction, and clustering tools that can be used to identify spatial domain. stlearn (2020) integrated gene expression, spatial data, and morphological features to efficiently identify spatial domains. SpaGCN (2021) combined gene expression and spatial data using GCN to identify spatial domains with consistent gene expression patterns. SEDR (2024) used a deep autoencoder to generate unsupervised spatial embeddings by learning gene representations and embedding spatial information with a variogram autoencoder. STAGATE (2022) fused spatial and gene expression information to identify spatial domains via an adaptive graph attention autoencoder. GraphST (2023) achieved spatial domain identification by integrating graph neural networks with self-supervised contrastive learning. Spatial-MGCN (2023) utilized a multi-view GCN encoder to identify spatial domains.

All benchmark methods were executed using their default parameters, with an equal number of clusters applied during the clustering process. Two widely used metrics (ARI [63] and NMI [64]) were used to evaluate the performance of each method.

### Evaluation metrics

Adjusted Rand Index (ARI) is a measure of clustering similarity that adjusts the Rand Index (RI) for chance. It quantifies how well the predicted clusters align with the ground truth labels while accounting for random cluster assignments. The ARI is calculated as follows:


$$\left\{ \begin{array}{c} ARI(W,V)=\frac{RI(W,V)-E\left[RI(W,V)\right]}{\max\left[RI(W,V)\right]-E\left[RI(W,V)\right]}\\ RI(W,V)=\frac{TP+TN}{TP+FP+FN+TN} \end{array}\right.$$ (16)

where TP is the number of true positives, TN is the number of true negatives, FP is the number of false positives, and FN is the number of false negatives.

Normalized Mutual Information (NMI) is a normalized version of Mutual Information (MI) that corrects for biases introduced by differing numbers of labels. It ensures that values range between 0 and 1, where 1 indicates perfect agreement between predicted clusters and ground truth labels, and 0 indicates no correlation. NMI is calculated as follows:


$$\left\{ \begin{array}{c} NMI(W,V)=\frac{MI(W,V)}{\sqrt{H(W)H(V)}}\\ MI(W,V)=\sum_{w\in U}\sum_{v\in V}P(w,v)\log\frac{P(w,v)}{P(w)P(v)}\\ H(W)=-\sum_{w\in W}P(w)\log P(w)\\ H(V)=-\sum_{v\in V}P(v)\log P(v) \end{array}\right.$$ (17)

Let W represent the set of clustering results, and V represent the set of ground truth. Then P(w) and P(v) denote the probabilities of the ground truth w and the clustering label v, while P(w,v) represents the probabilities of w and v occurring simultaneously. H(W) and H(V) denote the entropy of the clustering results and the true labels, respectively, reflecting the uncertainty in each label set.

## Supporting information

S1 Table Experimental results of SpaMWGDA and seven competing methods on the noisy DLPFC dataset. (DOCX)

S1 Fig Detailed results for all 12 slices in the DLPFC dataset. (TIF)
