# Correction: SpaMWGDA: Identifying spatial domains of spatial transcriptomes using multi-view weighted fusion graph convolutional network and data augmentation

**DOI:** 10.1371/journal.pcbi.1013877

**Published:** 2026-01-06

**Authors:** Lin Yuan, Boyuan Meng, Qingxiang Wang, Chunyu Hu, Cuihong Wang, De-Shuang Huang

There is an error in affiliation 4 for author Cuihong Wang. The correct affiliation 4 is: The Second Qilu Hospital of Shandong University, Shandong University, Jinan, China.
