# Supplementary material for: SpaMWGDA: Identifying spatial domains of spatial transcriptomes using multi-view weighted fusion graph convolutional network and data augmentation
Source: PLoS Comput Biol. 2025 Nov 12;21(11):e1013667. doi: 10.1371/journal.pcbi.1013667 (PMC12611167; doi:10.1371/journal.pcbi.1013667)
Supplement: S1 Table — (DOCX) [file pcbi.1013667.s001.docx]

**Supplementary Table 1.** Experimental results of SpaMWGDA and seven competing methods on the noisy DLPFC dataset.

| **20% Gaussian noise** | | | | | | | | |
| --- | --- | --- | --- | --- | --- | --- | --- | --- |
| **ARI** | **SpaMWGDA** | **Spatial-MGCN** | **STAGATE** | **SEDR** | **GraphST** | **SpaGCN** | **stLearn** | **Scanpy** |
| 151507 | 0.74 | 0.6 | 0.45 | 0.47 | 0.49 | 0.42 | 0.38 | 0.23 |
| 151508 | 0.68 | 0.52 | 0.43 | 0.48 | 0.38 | 0.42 | 0.34 | 0.25 |
| 151509 | 0.66 | 0.57 | 0.42 | 0.62 | 0.38 | 0.4 | 0.36 | 0.24 |
| 151510 | 0.53 | 0.53 | 0.42 | 0.44 | 0.48 | 0.3 | 0.27 | 0.21 |
| 151669 | 0.57 | 0.34 | 0.21 | 0.38 | 0.61 | 0.22 | 0.27 | 0.13 |
| 151670 | 0.51 | 0.43 | 0.26 | 0.26 | 0.49 | 0.2 | 0.28 | 0.15 |
| 151671 | 0.61 | 0.6 | 0.52 | 0.47 | 0.75 | 0.36 | 0.24 | 0.18 |
| 151672 | 0.83 | 0.6 | 0.43 | 0.47 | 0.46 | 0.51 | 0.25 | 0.16 |
| 151673 | 0.6 | 0.59 | 0.26 | 0.51 | 0.59 | 0.47 | 0.23 | 0.2 |
| 151674 | 0.58 | 0.58 | 0.34 | 0.49 | 0.28 | 0.41 | 0.27 | 0.19 |
| 151675 | 0.6 | 0.56 | 0.36 | 0.38 | 0.2 | 0.36 | 0.24 | 0.16 |
| 151676 | 0.57 | 0.53 | 0.43 | 0.31 | 0.19 | 0.41 | 0.23 | 0.16 |
| AVG. | 0.62 | 0.54 | 0.38 | 0.44 | 0.44 | 0.37 | 0.28 | 0.19 |
| **NMI** | **SpaMWGDA** | **Spatial-MGCN** | **STAGATE** | **SEDR** | **GraphST** | **SpaGCN** | **stLearn** | **Scanpy** |
| 151507 | 0.77 | 0.74 | 0.64 | 0.61 | 0.65 | 0.53 | 0.51 | 0.36 |
| 151508 | 0.72 | 0.64 | 0.59 | 0.54 | 0.52 | 0.53 | 0.53 | 0.35 |
| 151509 | 0.69 | 0.69 | 0.61 | 0.68 | 0.59 | 0.53 | 0.48 | 0.39 |
| 151510 | 0.66 | 0.67 | 0.61 | 0.64 | 0.64 | 0.48 | 0.45 | 0.34 |
| 151669 | 0.6 | 0.46 | 0.46 | 0.5 | 0.65 | 0.39 | 0.41 | 0.21 |
| 151670 | 0.61 | 0.57 | 0.46 | 0.36 | 0.53 | 0.38 | 0.4 | 0.24 |
| 151671 | 0.72 | 0.71 | 0.64 | 0.6 | 0.69 | 0.46 | 0.35 | 0.28 |
| 151672 | 0.8 | 0.72 | 0.6 | 0.61 | 0.64 | 0.59 | 0.39 | 0.24 |
| 151673 | 0.68 | 0.66 | 0.48 | 0.65 | 0.7 | 0.6 | 0.43 | 0.38 |
| 151674 | 0.66 | 0.69 | 0.49 | 0.66 | 0.46 | 0.49 | 0.41 | 0.32 |
| 151675 | 0.67 | 0.66 | 0.53 | 0.58 | 0.42 | 0.48 | 0.44 | 0.33 |
| 151676 | 0.67 | 0.65 | 0.58 | 0.51 | 0.42 | 0.53 | 0.39 | 0.28 |
| AVG. | 0.69 | 0.66 | 0.56 | 0.58 | 0.58 | 0.5 | 0.43 | 0.31 |
| **30% Gaussian noise** | | | | | | | | |
| **ARI** | **SpaMWGDA** | **Spatial-MGCN** | **STAGATE** | **SEDR** | **GraphST** | **SpaGCN** | **stLearn** | **Scanpy** |
| 151507 | 0.71 | 0.57 | 0.56 | 0.45 | 0.42 | 0.44 | 0.36 | 0.24 |
| 151508 | 0.65 | 0.58 | 0.44 | 0.31 | 0.52 | 0.39 | 0.38 | 0.25 |
| 151509 | 0.64 | 0.55 | 0.48 | 0.48 | 0.46 | 0.4 | 0.43 | 0.24 |
| 151510 | 0.51 | 0.56 | 0.38 | 0.42 | 0.32 | 0.42 | 0.31 | 0.22 |
| 151669 | 0.57 | 0.45 | 0.39 | 0.49 | 0.38 | 0.21 | 0.28 | 0.16 |
| 151670 | 0.48 | 0.45 | 0.34 | 0.57 | 0.53 | 0.33 | 0.17 | 0.16 |
| 151671 | 0.7 | 0.6 | 0.5 | 0.46 | 057 | 0.44 | 0.24 | 0.18 |
| 151672 | 0.84 | 0.84 | 0.54 | 0.62 | 0.52 | 0.36 | 0.25 | 0.18 |
| 151673 | 0.55 | 0.58 | 0.45 | 0.47 | 0.25 | 0.43 | 0.22 | 0.2 |
| 151674 | 0.57 | 0.55 | 0.36 | 0.47 | 0.26 | 0.39 | 0.36 | 0.19 |
| 151675 | 0.59 | 0.55 | 0.33 | 0.35 | 0.16 | 0.42 | 0.17 | 0.16 |
| 151676 | 0.57 | 0.49 | 0.5 | 0.37 | 0.19 | 0.39 | 0.37 | 0.16 |
| AVG. | 0.62 | 0.56 | 0.44 | 0.45 | 0.38 | 0.39 | 0.3 | 0.2 |
| **NMI** | **SpaMWGDA** | **Spatial-MGCN** | **STAGATE** | **SEDR** | **GraphST** | **SpaGCN** | **stLearn** | **Scanpy** |
| 151507 | 0.76 | 0.72 | 0.66 | 0.61 | 0.59 | 0.55 | 0.48 | 0.37 |
| 151508 | 0.72 | 0.67 | 0.61 | 0.43 | 0.61 | 0.5 | 0.5 | 0.35 |
| 151509 | 0.7 | 0.67 | 0.63 | 0.64 | 0.6 | 0.54 | 0.55 | 0.38 |
| 151510 | 0.69 | 0.67 | 0.57 | 0.59 | 0.52 | 0.5 | 0.41 | 0.35 |
| 151669 | 0.59 | 0.59 | 0.54 | 0.52 | 0.46 | 0.41 | 0.39 | 0.24 |
| 151670 | 0.62 | 0.54 | 0.48 | 0.59 | 0.59 | 0.45 | 0.3 | 0.25 |
| 151671 | 0.7 | 0.63 | 0.63 | 0.61 | 0.66 | 0.53 | 0.35 | 0.28 |
| 151672 | 0.81 | 0.79 | 0.65 | 0.6 | 0.63 | 0.45 | 0.37 | 0.25 |
| 151673 | 0.65 | 0.66 | 0.63 | 0.62 | 0.49 | 0.53 | 0.41 | 0.38 |
| 151674 | 0.65 | 0.66 | 0.5 | 0.61 | 0.42 | 0.49 | 0.46 | 0.32 |
| 151675 | 0.67 | 0.68 | 0.46 | 0.57 | 0.37 | 0.55 | 0.34 | 0.33 |
| 151676 | 0.67 | 0.61 | 0.63 | 0.56 | 0.42 | 0.47 | 0.54 | 0.29 |
| AVG. | 0.69 | 0.66 | 0.58 | 0.58 | 0.53 | 0.42 | 0.43 | 0.32 |
