# Supplementary figures and images for: SpaMWGDA: Identifying spatial domains of spatial transcriptomes using multi-view weighted fusion graph convolutional network and data augmentation
Source: PLoS Comput Biol. 2025 Nov 12;21(11):e1013667. doi: 10.1371/journal.pcbi.1013667 (PMC12611167; doi:10.1371/journal.pcbi.1013667)

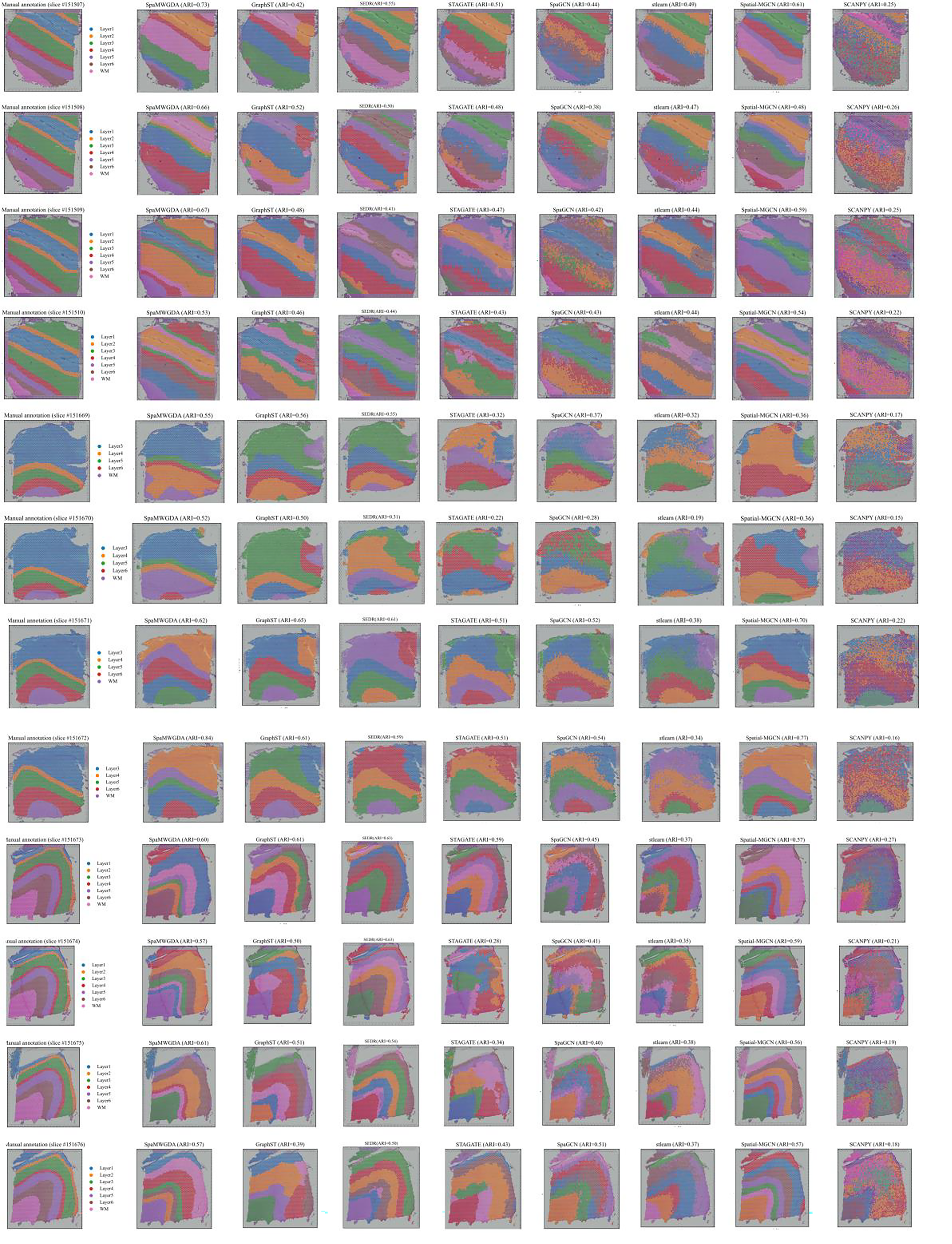

Supplement: S1 Fig — (TIF) [file pcbi.1013667.s002.tif]
